# Supplementary material for: Modelling Skylarks (Alauda arvensis) to Predict Impacts of Changes in Land Management and Policy: Development and Testing of an Agent-Based Model
Source: PLoS One. 2013 Jun 6;8(6):e65803. doi: 10.1371/journal.pone.0065803 (PMC3675089; doi:10.1371/journal.pone.0065803)
Supplement: Supporting Information S4 — The skylark ODdox as a zipped archive. (ZIP) [file pone.0065803.s004.zip › Skylark_ODdox/maperrormsg_8h.html]

ALMaSS Skylark ODdox: maperrormsg.h File Reference


ALMaSS Skylark ODdox
 2.0


- Main Page
- Related Pages
- Classes
- Files

- File List
- File Members

Classes |
Enumerations |
Variables

maperrormsg.h File Reference

|  |  |
| --- | --- |
| Classes | |
| class | MapErrorMsg |

|  |  |
| --- | --- |
| Enumerations | |
| enum | MapErrorState {     WARN\_BUG, WARN\_FATAL, WARN\_UNDEF, WARN\_FILE,     WARN\_MSG, WARN\_TRIVIAL, WARN\_ALL   } |

|  |  |
| --- | --- |
| Variables | |
| class MapErrorMsg \* | g\_msg |

---

## Enumeration Type Documentation

enum MapErrorState

**Enumerator:**
:   |  |  |
    | --- | --- |
    | *WARN\_BUG* |  |
    | *WARN\_FATAL* |  |
    | *WARN\_UNDEF* |  |
    | *WARN\_FILE* |  |
    | *WARN\_MSG* |  |
    | *WARN\_TRIVIAL* |  |
    | *WARN\_ALL* |  |

{

WARN\_BUG,

WARN\_FATAL,

WARN\_UNDEF,

WARN\_FILE,

WARN\_MSG,

WARN\_TRIVIAL,

WARN\_ALL

} MapErrorState;

---

## Variable Documentation

class MapErrorMsg\* g\_msg

Referenced by Landscape::AddBeetleBanks(), LE\_TypeClass::BackTranslateEleTypes(), LE\_TypeClass::BackTranslateVegTypes(), Landscape::BorderAdd(), Landscape::BorderNeed(), Skylark\_Population\_Manager::BreedingPairsOutput(), Skylark\_Population\_Manager::BreedingSuccessProbeOutput(), Landscape::ChangeMapMapping(), Farm::CheckRotationManagementLoop(), FarmManager::ConnectFarm(), FarmManager::CreateFarms(), CropRotation::CropRotation(), Calendar::DayLength(), RodenticideManager::DoPlaceBait(), Landscape::DumpMap(), Landscape::DumpMapGraphics(), SkTerritories::DumpMapGraphics(), Landscape::DumpMapInfoByArea(), Configurator::DumpSymbols(), Landscape::DumpTreatCounters(), Landscape::EventDump(), Landscape::EventtypeToString(), Configurator::ExtractString(), probe\_data::FileAppendOutput(), CropData::FindCropNum(), Skylark\_Population\_Manager::FledgelingProbeOutput(), Landscape::ForceArea(), RasterMap::Get(), CropRotation::GetFirstCrop(), CropRotation::GetNextCrop(), Landscape::IncTreatCounter(), MovementMap::Init(), Farm::InitiateManagement(), UserDefinedFarm::InvIntPartition(), Landscape::Landscape(), Farm::LeSwitch(), Farm::Management(), Landscape::NewElement(), probe\_data::OpenFile(), probe\_data::OpenForAppendToFile(), Skylark\_Population\_Manager::OpenTheBreedingPairsProbe(), Skylark\_Population\_Manager::OpenTheBreedingSuccessProbe(), Population\_Manager::OpenTheCIPEGridOutputProbe(), Population\_Manager::OpenTheMonthlyRipleysOutputProbe(), Population\_Manager::OpenTheReallyBigProbe(), Population\_Manager::OpenTheRipleysOutputProbe(), Landscape::OrchardBorderAdd(), Configurator::ParseCfgLine(), Pesticide::Pesticide(), Landscape::PolysDump(), Landscape::PolysValidate(), Landscape::PolytypeToString(), SkTerritories::PreFillQualGrid(), RasterMap::RasterMap(), VegElement::ReadBugPercentageFile(), Landscape::ReadPolys(), Farm::ReadRotation(), Configurator::ReadSymbols(), Farm::RemoveField(), Pesticide::SavePPM(), Configurator::SetCfgBool(), Configurator::SetCfgFloat(), Configurator::SetCfgGatekeeper(), Configurator::SetCfgInt(), Configurator::SetCfgStr(), CropData::SetVegNum(), Configurator::ShowIdType(), Skylark\_Female::st\_Finding\_Territory(), Skylark\_Female::st\_Laying(), Skylark\_Female::st\_MakingNest(), Farm::TranslateCropCodes(), LE\_TypeClass::TranslateEleTypes(), LE\_TypeClass::TranslateVegTypes(), Landscape::UnsprayedMarginAdd(), UserDefinedFarm::UserDefinedFarm(), Landscape::VegDump(), CropData::VegTypeToCurveNum(), Landscape::VegtypeToString(), Landscape::Warn(), Weather::Weather(), and Landscape::~Landscape().


- CJT
- MSVC
- ALMaSS Working Source
- Landscape
- maperrormsg.h
- Generated on Thu Jan 10 2013 13:15:35 for ALMaSS Skylark ODdox by
   1.8.1.1
